# Structural basis for antibody recognition of the NANP repeats in *Plasmodium falciparum* circumsporozoite protein

**DOI:** 10.1073/pnas.1715812114

**Published:** 2017-11-14

**Authors:** David Oyen, Jonathan L. Torres, Ulrike Wille-Reece, Christian F. Ockenhouse, Daniel Emerling, Jacob Glanville, Wayne Volkmuth, Yevel Flores-Garcia, Fidel Zavala, Andrew B. Ward, C. Richter King, Ian A. Wilson

**Affiliations:** ^a^Department of Integrative Structural and Computational Biology, The Scripps Research Institute, La Jolla, CA 92037;; ^b^PATH’s Malaria Vaccine Initiative, PATH Center for Vaccine Innovation and Access, Washington, DC 20001;; ^c^Atreca Inc., Redwood City, CA 94063;; ^d^Department of Microbiology and Immunology, Stanford University, Stanford, CA 94305;; ^e^Malaria Research Institute, Johns Hopkins Bloomberg School of Public Health, Baltimore, MD 21205;; ^f^The Skaggs Institute for Chemical Biology, The Scripps Research Institute, La Jolla, CA 92037

**Keywords:** malaria, circumsporozoite protein, antibodies, X-ray crystallography, EM

## Abstract

Acquired resistance against antimalarial drugs has further increased the need for an effective malaria vaccine. The current leading candidate, RTS,S, is a recombinant circumsporozoite protein (CSP)-based vaccine against *Plasmodium falciparum* that contains 19 NANP repeats followed by a thrombospondin repeat domain. Although RTS,S has undergone extensive clinical testing and has progressed through phase III clinical trials, continued efforts are underway to enhance its efficacy and duration of protection. Here, we determined that two monoclonal antibodies (mAbs 311 and 317), isolated from a recent controlled human malaria infection trial exploring a delayed fractional dose, inhibit parasite development in vivo by at least 97%. Crystal structures of antibody fragments (Fabs) 311 and 317 with an (NPNA)_3_ peptide illustrate their different binding modes. Notwithstanding, one and three of the three NPNA repeats adopt similar well-defined type I β-turns with Fab311 and Fab317, respectively. Furthermore, to explore antibody binding in the context of *P. falciparum* CSP, we used negative-stain electron microscopy on a recombinant shortened CSP (rsCSP) construct saturated with Fabs. Both complexes display a compact rsCSP with multiple Fabs bound, with the rsCSP–Fab311 complex forming a highly organized helical structure. Together, these structural insights may aid in the design of a next-generation malaria vaccine.

## Significance

The *Plasmodium falciparum* circumsporozoite protein (CSP) has been studied for decades as a potential immunogen, but little structural information is available on how antibodies recognize the immunodominant NANP repeats within CSP. The most advanced vaccine candidate is RTS,S, which includes multiple NANP repeats. Here, we analyzed two functional antibodies from an RTS,S trial and determined the number of repeats that interact with the antibody Fab fragments using isothermal titration calorimetry and X-ray crystallography. Using negative-stain electron microscopy, we also established how the antibody binds to the NANP repeat region in a recombinant CSP construct. The structural features outlined here provide a rationale for structure-based immunogen design to improve upon the efficacy of the current RTS,S vaccine.

Malaria remains one of the world’s most important public health challenges with an estimated 212 million cases and 429,000 deaths in 2015 (1). Although these numbers have been declining over the past 15 years, further progress is being thwarted by the appearance of drug-resistant parasite strains and insecticide-resistant mosquitoes (2). To promote malaria elimination and eradication campaigns, the WHO has prioritized development of a malaria vaccine (2). The most advanced vaccine at present is RTS,S, a *Plasmodium falciparum* circumsporozoite protein (CSP)-based vaccine. In a phase III clinical trial, RTS,S reduced the incidence of clinical malaria by ∼51% (over the first 14 mo) in children who were 5–17 mo old when they received the first of three doses (3). Protection was highest immediately after vaccination, waned over time, and was enhanced by a fourth dose 18 mo after dose 3 (4). Over 48 mo (median), vaccine efficacy was 28% following three doses and 36% after four doses (5). Thus, this vaccine represents a major advance for the malaria field, particularly with recent reports of drug-resistant *P. falciparum* in South East Asia (6). An important objective is to improve and extend the efficacy of antimalarial vaccines by exploiting knowledge of how protective antibodies recognize CSP and how vaccine-induced immunity impacts the parasite life cycle.

RTS,S targets the pre-erythrocytic stage of the *P. falciparum* life cycle, in which sporozoites are introduced into humans from the mosquito and then migrate to the liver. The sporozoite is coated with CSP, which is required for sporozoite development in infected mosquitos and for adhesion and invasion of hepatocytes in humans (7–9). CSP is comprised of an immunogenic central repeat region flanked by two conserved regions, the N-terminal domain and the C-terminal α-thrombospondin repeat (αTSR) domain with a glycosylphosphatidylinositol (GPI) anchor for attachment to the sporozoite membrane (10–12). The repeat region of the *P. falciparum* 3D7 strain CSP consists of four NVDP and 38 NANP repeats (13), although the exact number can differ per strain (14, 15), and is predicted to be structurally disordered (16). The first three NVDP repeats are interspersed between the first three NANP repeats, while a fourth is located after the first 20 NANP repeats.

CSP-based vaccine efforts, including the RTS,S vaccine, have focused primarily on these immunogenic repeats. RTS,S contains 19 NANP repeats and the C-terminal αTSR domain without the GPI anchor that are fused to hepatitis B viral surface protein, such that virus-like particles are formed (17, 18). When combined with the adjuvant AS01, very robust immune responses against the repeat region can be obtained shortly following vaccination with serum antibody concentrations of over 100 μg/mL (19). Clinical trials employing the human challenge model indicate that protection may be improved by modulating the dose and administration schedule (20, 21). In a recent phase IIa RTS,S/AS01B controlled human malaria infection (CHMI) trial, the standard administration regimen of three monthly vaccinations resulted in 63% protection, while vaccination with a delayed fractional dose showed 87% protection (21). Over 100 antibodies to the NANP repeats and 20 against the C-terminal region of CSP have been identified by sequencing plasmablast mRNAs derived from both the standard and the delayed-fractional dose arms and from protected and nonprotected individuals. In all groups within the trial, two related IGHV3 heavy-chain variable regions (HV3-30 and HV3-33) predominate in the antibody sequences.

Here, we sought to understand the binding and specificity of mAbs derived from a protected individual within the fractionated dose administration arm of the CHMI trial. Our goal was to understand the nature and specificity of RTS,S-elicited Abs at the molecular level to guide structure-aided vaccine design. We selected two individual NANP-specific human mAbs, 311 and 317 [HV3-33 and HV3-30, with different IGLV1 light chains (LV1-40 and KV1-5, respectively)] for structural study. Both antibodies inhibited infection in vivo using transgenic *Plasmodium berghei* in which its CSP protein was replaced with *P. falciparum* CSP. X-ray structures were determined for the NANP repeats in complex with antibody fragments (Fabs) derived from mAbs 311 and 317, and negative-stain electron microscopy (nsEM) evaluated how the NANP repeats were recognized in the context of CSP. Despite the intrinsic disorder of the NANP repeat region, we show that these Fabs recognize distinct secondary structures within the repeat region that likely are in equilibrium with its generally unfolded state in solution or that possibly represent different states on CSP. Furthermore, a supramolecular structure is formed when the antibodies are bound to a recombinant form of CSP. These important structural features provide a platform to redesign the NANP repeat region in the current RTS,S vaccine to improve the efficacy and duration of the immune response.

## Results

### In Vivo Evaluation of Protection.

**Fig. 1.**
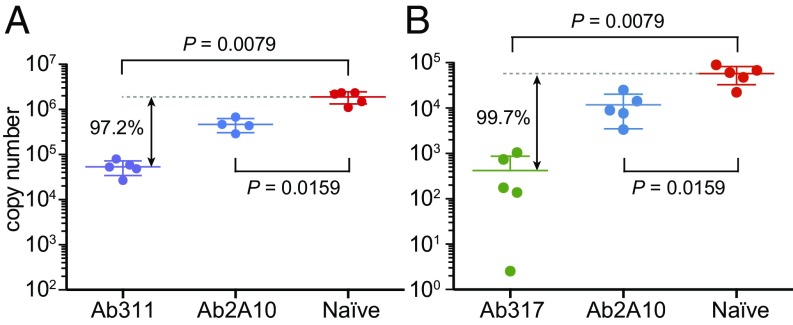
Antibody inhibition of malaria infection in mice. Parasite liver load 40 h post challenge with a chimeric *P. berghei* strain as assessed by qPCR for *P. berghei*-specific 18S rRNA after administration of Ab311 (100 μg) (*A*) and Ab317 (300 μg) (*B*). Significant protection is observed compared with naive mice, with 97.2% and 99.7% inhibition of parasite development for Ab311 and Ab317, respectively, while a previously reported antibody, 2A10 (300 μg dose) (24) showed only 75–82% inhibition of the parasite liver load. The *P* values were determined using the Mann–Whitney *U* test. Only four data points are available for 2A10 in *A* because one mouse died.

Mice were administered mAbs 311 and 317 at a dose of 100 and 300 μg, respectively, and then were challenged 5–10 min later with *P. berghei* that was engineered to express full-length CSP from the *P. falciparum* 3D7 strain (22). The parasite liver load was assessed in the mice at 40 h postchallenge by qPCR for *P. berghei*-specific 18S rRNA (23). The mAbs 311 and 317 inhibited parasite development by at least 97% (97.2% and 99.7%, respectively) (Fig. 1), compared with only 75–82% inhibition for a previously reported anti-NANP antibody, 2A10 (300 μg dose) (24). Thus, mAbs 311 and 317 represent functional antibodies that are associated with strong protection.

### Epitope Mapping for Fab Fragments of 317 and 311 mAbs.

**Fig. 2.**
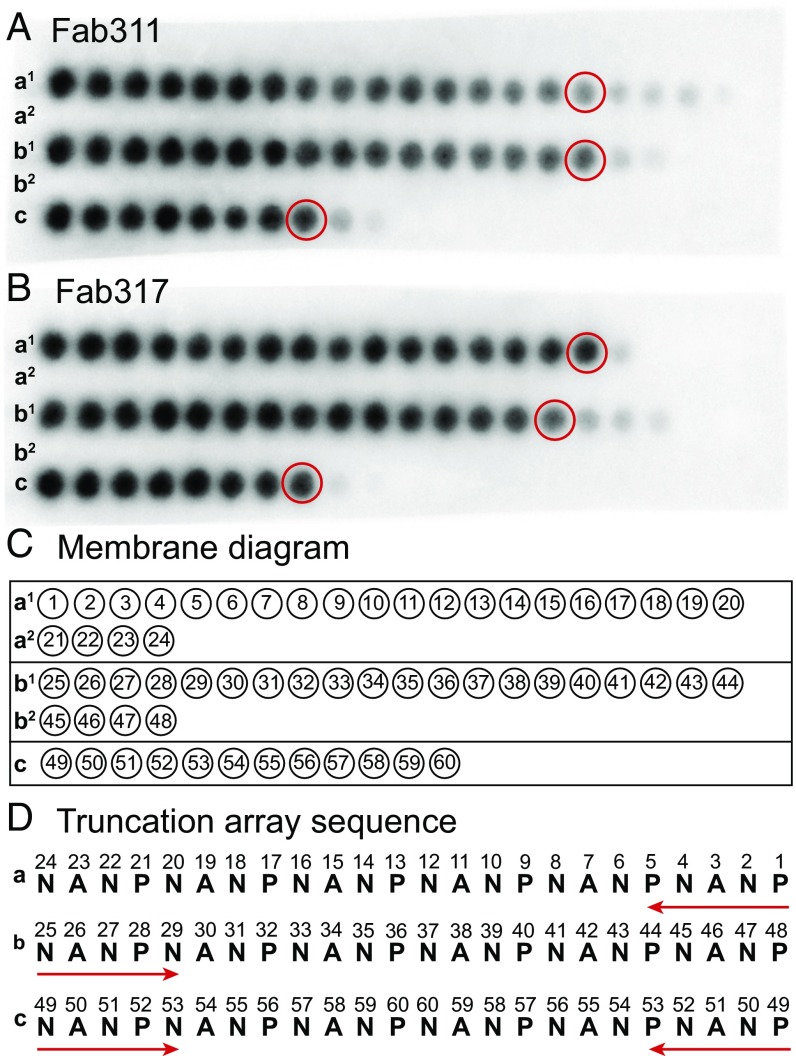
Epitope mapping using truncation peptide arrays. (*A* and *B*) The PepSpot membrane is shown for Fab311 (*A*) and Fab317 (*B*) and consists of five rows of the spotted peptides (a^1^, a^2^, b^1^, b^2^, and c). Dark spots indicate strong Fab binding. (*C* and *D*) Schematic of the location of the peptide spots on the membrane. The numbers within the circles refer to the numbers in the truncation array sequence (*D*). Rows a^1^ and a^2^ correspond to a truncation array starting from the C terminus of the (NANP)_6_ peptide, rows b^1^ and b^2^ are truncations from the N terminus, and row c represents truncations from both the N terminus and C terminus simultaneously. The peptides that appear to have the minimal number of repeats for strong Fab binding are circled in red in *A* and *B*.

To determine the minimal NANP repeat sequence for binding, a customized peptide array (PepSpot) was designed that contains a series of truncated peptides derived from a 24-mer (NANP)_6_ peptide, which were spot-synthesized onto a cellulose membrane. Fab311 and Fab317 bind strongly to 10 or more amino acids regardless of the truncation approach, suggesting that a minimum epitope for strong binding consists of around 2.5 NANP repeats (Fig. 2). Additional weak spots for Fab311 indicate it can still bind shorter peptides, down to 1.5 NANP repeats (Fig. 2), whereas Fab317 requires a minimum of two NANP repeats. We therefore selected a 12-mer Ac-NPNANPNANPNA-NH_2_ peptide, (NPNA)_3_, for structural and binding studies.

### Peptide Affinity Measurements.

The (NPNA)_3_ peptides bind strongly to the antibodies with *K*_d_s of 305 ± 60 nM for Fab311 and 78 ± 16 nM for Fab317 (Table 1 and Fig. S1). To determine the minimal epitope for optimal binding, binding affinities for an (NPNA)_2_ peptide were measured. The *K*_d_ for (NPNA)_2_ binding to Fab311 (296 ± 19 nM) is almost identical to that of the (NPNA)_3_ peptide, whereas the *K*_d_ for (NPNA)_2_ binding to Fab317 increased 2.2-fold (173 ± 8 nM). Although NVDP repeats were not present in the RTS,S vaccine, we decided to test whether such a sequence could be accommodated by the antibodies, because they are present in the wild-type CSP. Fab311 has approximately fivefold lower affinity for peptides Ac-NPNVDPNANPNV-NH_2_ and Ac-DPNANPNVDPNA-NH_2_ (*K*_d_s of 1.79 ± 0.19 μM and 1.37 ± 0.39 μM, respectively), whereas Fab317 bound to these same peptides with substantially lower (*K*_d_ of 12.09 ± 2.49 μM) and fourfold higher (*K*_d_ of 0.45 ± 0.07 μM) affinity, respectively (Table 1 and Fig. S2).

**Table 1.** Dissociation constants for NANP/NVDP repeat-containing peptides obtained from ITC affinity measurements

Binding parameter	(NPNA)_3_	(NPNA)_2_	NPNVDPNANPNV	DPNANPNVDPNA
Fab311				
No. of sites	0.74 ± 0.02	1.50 ± 0.08	1.24 ± 0.02	1.20 ± 0.03
*K*_d_, μM	0.305 ± 0.060	0.296 ± 0.019	1.787 ± 0.189	1.368 ± 0.390
∆H, cal/mol	−29,823 ± 261	−16,363 ± 261	−18,657 ± 378	−20,200 ± 1,078
∆S, cal^−1^⋅mol^−1^⋅degree^−1^	−70 ± 1	−25 ± 1	−36 ± 2	−41 ± 4
Fab317				
No. of sites	1.27 ± 0.02	1.41 ± 0.05	1.72 ± 0.13*	1.64 ± 0.05
*K*_d_, μM	0.078 ± 0.016	0.173 ± 0.008	12.091 ± 2.488*	0.448 ± 0.068
∆H, cal/mol	−15,700 ± 225	−12,090 ± 221	−9,657 ± 426*	−9,387 ± 152
∆S, cal^−1^⋅mol^−1^⋅degree^−1^	−20 ± 1	−10 ± 1	−10 ± 2*	−2 ± 1

All values are the average and SD of triplicate experiments except for those with an asterisk (*), which are from duplicate experiments.

### Crystal Structures of Fab311 and Fab317 in Complex with the (NPNA)_3_ Peptide.

Fab311 and Fab317 crystals with the (NPNA)_3_ peptide diffracted to 2.1 Å and 2.4 Å, respectively. Fab311–peptide crystallized in space group P2_1_2_1_2_1_ with a single complex in the crystal asymmetric unit (asu), whereas Fab317–peptide crystallized in space group P2_1_ with two complexes in the asu. The Fabs were numbered with the Kabat system, and the (NPNA)_3_ peptides were numbered from 2 to 13, with the N-terminal acetyl group and C-terminal NH_2_ group being assigned as residues 1 and 14, respectively.

**Fig. 3.**
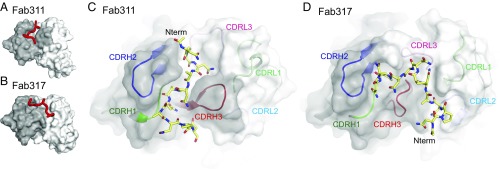
Crystal structures of (NPNA)_3_ peptides in complex with Fab311 and Fab317. (*A* and *B*) Surface representation of the variable domains of Fab311 (*A*) and Fab317 (*B*) with the (NPNA)_3_ peptide represented by a red tube. The heavy- and light-chain variable domains are colored dark and light gray, respectively. (*C* and *D*) Paratope representation for Fab311 (*C*) and Fab317 (*D*) with a transparent dark gray surface for the heavy chain and a transparent light gray surface for the light chain. The underlying CDR loops are shown in cartoon representation and are colored green (H1), blue (H2), red (H3), light green (L1), light blue (L2), and pink (L3). The (NPNA)_3_ peptide is shown in stick representation (yellow carbons). The N terminus of each peptide is indicated (Nterm).

**Fig. 4.**
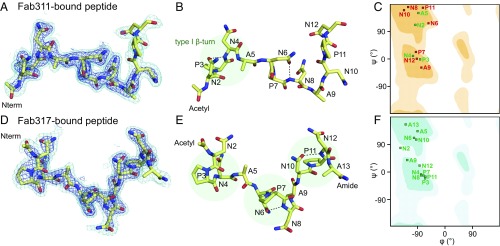
Structural analysis of antibody-bound peptides. (*A* and *D*) 2Fo-Fc electron density maps contoured at 2.0σ (blue) and 0.8σ (cyan) for peptide bound to Fab311 (*A*) and Fab317 (*D*). The peptide is shown in stick representation (yellow carbons). (*B* and *E*) Type I β-turns are highlighted by transparent green circles for peptide bound to Fab311 (*B*) and Fab317 (*E*). Intrapeptide hydrogen bonds that emulate a pseudo 3_10_ turn between the first Asn sidechain and amide backbone of the third residue in the turn are shown as black dashed lines. (*C* and *F*) Ramachandran plots for the dihedral angles of Fab311-bound peptide (*C*) and Fab317-bound peptide (*F*). Residues that have typical dihedral angles indicative of canonical NPNA type I β-turns are colored green; otherwise they are colored red. The β-sheet region is in the dark shaded region of the plot in the upper left quadrant, and the α-helical region is in the central region on the left around ψ of −30°. The Fab311-bound and Fab317-bound peptides have one and three canonical type I β-turns, respectively.

The electron density for the peptides in both complexes is generally well defined. The (NPNA)_3_ peptide forms largely extended structures in both antibody-binding sites with kinks in the peptide that arise from defined secondary structures (Fig. 3). Dihedral angle analysis shows that type I β-turns (residues i to i + 3) are found for the NPNA repeats, which are stabilized by hydrogen bonding of the first Asn side chain (residue i) to the backbone amide of the next Asn (i + 2) (Fig. 4). The N-terminal NPNA repeats of both peptides adopt type I β-turns. While this cadence of type I β-turns continues in the Fab317-bound peptide, the Fab311-bound peptide maintains a more extended structure for the second and third NPNA repeats, both of which deviate from canonical type I β-turns (Fig. 4 *B* and *E*). Inspection of 2Fo-Fc maps contoured at 2.0σ and 0.8σ (Fig. 4 *A* and *D*) reveals that the electron density for the peptide bound to Fab311 is weaker after Ala9 and absent for the C-terminal Ala13. In contrast, the (NPNA)_3_ peptide bound to Fab317 is well defined for all peptide residues except Ala13.

The peptide interaction with the complementarity-determining region (CDR) loops is very different in each structure, however. In Fab311, the peptide resides in a groove that runs parallel to the heavy and light chains. The peptide N-terminal region is located between CDR H2 and CDR L3, and the peptide then threads between CDR H2, CDR H3, and CDR H1. The Fab311 CDR H3 (12 residues) helps form the groove in which the peptide resides (Fig. 3 *A* and *C*). The heavy chain contributes 80.2% of the buried surface area (BSA) (574 Å^2^ on the Fab and 520 Å^2^ on the peptide) from CDR H1 (17.4%), H2 (30.5%), and H3 (32.3%), while the light chain contributes only 19.3%, mainly CDR L3 (17.3%). The remaining 0.5% is contributed by a heavy-chain framework residue. In Fab317, the peptide binds in a more perpendicular mode compared with Fab311 and occupies a relatively shallow binding pocket that initially starts between the light and heavy chains and then bends sharply and traverses from the light to the heavy chain (Fig. 3 *B* and *D*). The heavy chain contributes 58.2% of the BSA from CDR H1 (5.4%), H2 (21.7%), and H3 (31.1%), while the light chain contributes 39.3% from CDR L1 (10.9%), L2 (7.7%), and L3 (20.7%), with the remaining 2.5% from a light-chain framework residue. The BSAs on Fab317 and (NPNA)_3_ peptide are 533 Å^2^ and 580 Å^2^, respectively. Since CDR H3 and CDR L3 contribute ∼30% and ∼20%, respectively, of the BSA for both Fab311 and Fab317, differences in the binding mode arise mainly from different interactions with light- and heavy-chain CDR1 and CDR2.

Hydrogen-bonding networks and amino acid BSAs highlight additional differences. Most of the hydrogen bonds between Fab311 and peptide are made with the C-terminal half of the peptide (Table S2), although its N-terminal region (Pro3, Asn4, and Ala5) engages Fab311 through a hydrogen-bonding network that includes four interfacial water molecules (Fig. S3). Furthermore, Pro3 and Pro7 of the (NPNA)_3_ peptide are buried in the Fab311 paratope groove with BSAs of 76 Å^2^ and 75 Å^2^, respectively, and engage in CH/π interactions with Phe58^H^ and Trp52^H^ (Fig. S3). The peptide-binding site on Fab317 does not contain any observable interfacial water molecules. Hydrogen-bonding partners with Fab317 are evenly distributed throughout the peptide (Asn4, Asn6, Ala9, Pro11, and Asn12) (Table S2). In contrast, Pro3 and Pro7 have small BSAs of 22 Å^2^ and 10 Å^2^, respectively. Pro11 has a larger BSA of 78 Å^2^, compared with only 7 Å^2^ in the Fab311–peptide complex.

### nsEM of CSP with Protective Antibodies.

**Fig. 5.**
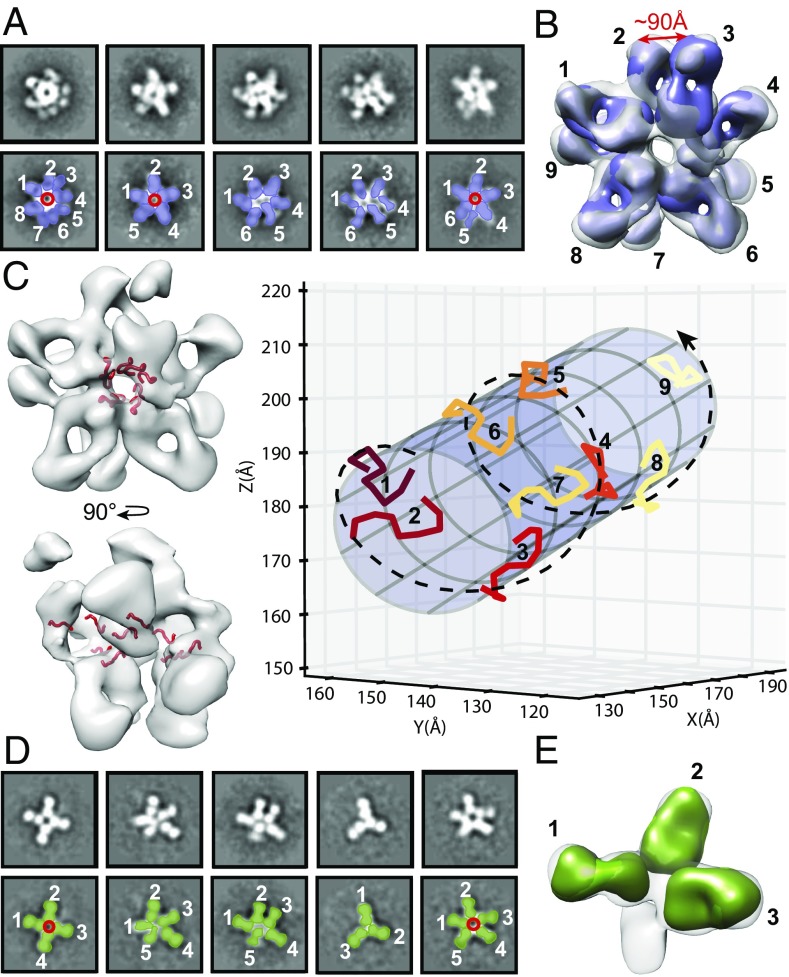
nsEM for rsCSP bound to Fab311 and Fab317. (*A* and *D*) Five selected representative class averages for the rsCSP–Fab311 (*A*) and rsCSP–Fab317 (*D*) complexes, false colored to show the location of rsCSP (red), Fab311 (purple), and Fab317 (green). Fabs are labeled by number in white. (*B*) The refined 3D model confirmed the presence of multiple densities for Fab311, for which a total count of nine Fab311s could be observed at the low threshold level. The distance between the heavy-chain C termini of Fab311 nos. 2 and 3 was measured at 90 Å, which could be accommodated in an IgG. (*C*) The (NPNA)_3_ peptide (red) from the Fab311 crystal structure was docked into the 3D model (*Left*) and revealed a helical shape looking into the central hole of the rsCSP complex and along its length (*Right*). The docked peptides in the nsEM map for the rsCSP–Fab311 complex were fitted to a cylinder with a radius of 15.2 Å. The peptides are colored using a color progression, and the helical organization is shown by the dashed spiral line. (*D* and *E*) Reaching convergence for the rsCSP–Fab317 complex (*E*) was difficult due to the various stoichiometries seen in the 2D class averages (*D*). The unmodeled blob may be a remnant of a fourth Fab fragment that is present in some of the complexes.

Single-particle nsEM was then utilized to visualize the stoichiometry and overall molecular organization of the CSP–Fab complexes using an engineered recombinant version of CSP with a reduced number of NANP/NVDP repeats [hereafter, “recombinant shortened CSP” (rsCSP): 19/3 repeats instead of 38/4 for the *P. falciparum* 3D7 strain]. Visual inspection of the 2D class averages for the Fab311–rsCSP complex indicate a binding stoichiometry of five or more per rsCSP molecule (Fig. 5*A*), while in Fab317–rsCSP up to five Fabs are bound (Fig. 5*D* and Fig. S4). Further 3D classification and refinement of the Fab311–rsCSP complex data using C1 symmetry revealed up to nine Fabs bound per rsCSP molecule (Fig. 5*B*). However, at lower threshold values of the negative-stain map, additional Fab densities became visible (threshold values of 0.274, 0.223, and 0.179 reveal five, eight, and nine Fabs per complex, respectively), perhaps as a result of substoichiometric complexes that were averaged with the rest of the data. Docking the Fab311–(NPNA)_3_ crystal structures into the EM density map shows that the peptides and, by extension, rsCSP adopt an extended, left-handed, helical conformation with a fitted radius of ∼15 Å when bound to this antibody (Fig. 5*C* and Fig. S4*C*). The Ala9 and Asn2 C_α_ atoms in the crystal structure of two adjacent (NPNA)_3_ peptides are, on average, 13 ± 2 Å apart when fitted into the EM reconstruction, and about five peptides complete one turn around the helical axis. In total, we observe two full turns that comprise a distance of 97 Å between the N termini of the first and last peptides in the helical structure (Fig. 5 *B* and *C*). Notably, there is little or no density in the map that would correspond to the N- or C-terminal domains of the rsCSP, which may be the result of intrinsic disorder or flexibility relative to the ordered NANP–Fab–bound repeats. In contrast, the Fab317–rsCSP complex had fewer Fabs bound, and therefore a smaller portion of the structure was resolved. While 2D classification demonstrates up to five Fabs bound, the 3D refinement converged to three Fabs bound per rsCSP, which may be attributable to various stoichiometries, as seen in the class averages (Fig. 5 *D* and *E*). Similar to Fab311–rsCSP, the Fabs in the Fab317–rsCSP complex are in very close proximity. The differences in overall shape between the two complexes indicate some plasticity of rsCSP. We do not know whether its conformation is influenced by Fab binding, as the unliganded CSP structure is not known.

## Discussion

### The Minimal Epitope for Binding Consists of Two or Three NPNA Repeats.

The buried surface areas for the (NPNA)_3_ peptides bound to Fab311 and Fab317 indicate that all residues are engaged in Fab contacts except for Ala13 with Fab311 (Fig. S5). However, the main epitope of the Fab311-bound peptide is likely smaller than three NPNA repeats, as the last four residues have much weaker electron density and are likely more disordered (Fig. 4*A*). PepSpot epitope and isothermal titration calorimetry (ITC) affinity measurements (Fig. 2 and Table 1) confirm the hypothesis that the minimum epitope for optimal binding is two and three NPNA units for Fab311 and Fab317, respectively. These results are consistent with a previous study in which (NANP)_3_ peptides were able to efficiently inhibit binding of anti-NANP mAbs to *P. falciparum* sporozoites (25).

### NPNA Repeats Can Adopt Type I β-Turns in Solution and in Complex with Antibody.

**Fig. 6.**
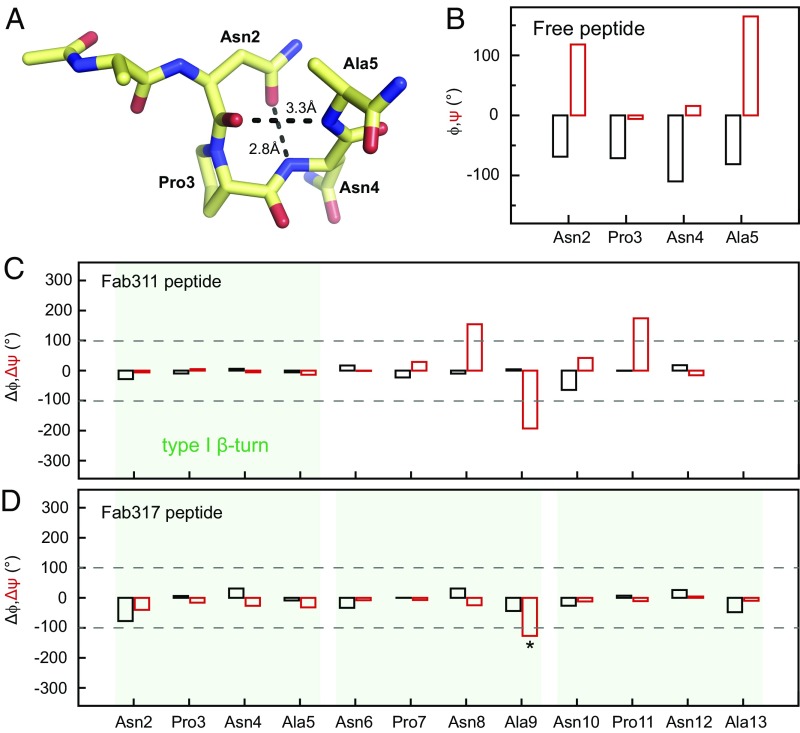
Comparison of dihedral angles shows similarities between the bound and free peptides. (*A*) X-ray structure of the free ANPNA peptide shows a type I β-turn in which the Asn2 (residue i) OD1 also hydrogen bonds to the backbone amide of Asn4 (i + 2) (27). (*B*) Plot of the dihedral angles for the NPNA unit in the ANPNA X-ray structure; ϕ and ψ are shown in black and red, respectively. (*C* and *D*) Plots of the dihedral angle differences between each of the NPNA units for the peptide bound to Fab311 (*C*) or Fab317 (*D*) and the NPNA unit of the free peptide; Δϕ and Δψ are shown in black and red, respectively. Type I β-turns are highlighted by transparent green boxes. The dihedral angle differences are relatively small within each NPNA type I β-turn, except for Ala9 Δψ in the Fab317 peptide (asterisk). This deviation from the NPNA type I β-turn in solution reflects a change in direction at the end of the NPNA repeat rather than a disruption of the canonical type I β-turn.

Previous studies on the NANP repeats focused on the unbound state using NMR spectroscopy (26) and X-ray crystallography (27) in which the NPNA units (residues i to i + 3) within NANP peptides of different lengths were in a dynamic equilibrium between a disordered state and an ordered type I β-turn. The highly populated type I β-turn is stabilized by a hydrogen bond between the side-chain of Asn (residue i) and the backbone amide of the next Asn (residue i + 2). Our crystal structures reveal that the bound peptides also adopt NPNA type I β-turns (Fig. 4). Specifically, peptides bound to Fab311 and Fab317 both have their N-terminal NPNA repeat in a canonical type I β-turn. The Fab317-bound peptide continues to display type I β-turns in a NPNA-like cadence, whereas the additional NPNA repeats in the Fab311-bound peptide have dihedral angles that deviate from a canonical type I β-turn (Fig. 4 *C* and *F*). Comparison of the dihedral angles for each NPNA repeat in our peptide to the ANPNA crystal structure (27) establishes that the type I β-turns in our Fab-bound peptides are nearly identical in conformation (Fig. 6). The peptide structures bound to Fabs also agree with NMR solution experiments that the CSP repeat region follows a cadence of NPNA repeats with each repeat having a high probability of forming a type I β-turn (26). Although the central NPNA repeat in the peptide bound to Fab311 does not form a canonical type I β-turn, the Asn6 side chain still hydrogen bonds to the Asn8 backbone amide (Fig. 4*B*). Interestingly, the relative orientation of this central NPNA repeat to the heavy-chain variable CDR loops is similar to that of the third NPNA repeat in the Fab317-bound peptide, which is expected since their heavy-chain germline genes are closely related (Fig. S6). Furthermore, a high Pearson correlation coefficient between the BSAs of the backbone and side-chain atoms for the second NPNA repeat of the Fab311 (NPNA)_3_ peptide with the third NPNA unit of the Fab317 (NPNA)_3_ peptide indicates a striking similarity in their binding mode (Fig. S7). These results are corroborated by similar hydrogen-bond networks for these residues, although the donor/acceptor residues on the Fab fragment are different (Table S2). Thus, elicitation of these antibodies may have originated by recognition of a single type I β-turn from which the antibodies independently evolved to increase the number of contacts and thus their affinity for the peptide repeats.

### The CSP Antigen Adopts a Collapsed Conformation in Its Antibody-Bound Form.

To date, the handful of studies that have attempted to elucidate the structure of full-length CSP, suggest that CSP exists in two conformations: open and collapsed (28, 29). CSP is speculated to be present in the collapsed form on the sporozoite surface during migration to the liver and in the open form when it fully extends to engage the host cell receptor for entry (28). However, determining the structure of full-length CSP has been challenging, which is not surprising since ∼75% of the CSP sequence, including the entire central NANP repeat region and part of the N-terminal domain, is predicted to be disordered (16). Here, we were able to obtain high-quality nsEM images for rsCSP saturated with Fab311 or Fab317. Despite the proposed intrinsic disorder of free CSP, both complexes display a collapsed state of the antigen with closely apposed Fabs observed in the EM projection. The rsCSP–Fab311 complex, in particular, adopts a large supramolecular structure that appears to form a spiral with the Fabs radiating tangentially from the surface of the CSP repeats (Fig. 5 *B* and *C*). Many questions remain as to whether this same structure is formed on the sporozoite surface or whether antibodies help induce this particular conformation. The C termini of the heavy chains of Fab311 are 90 Å apart (Fig. 5*B*), suggesting that an individual IgG is capable of binding to two adjacent epitopes within a single rsCSP antigen. Alternatively, given the avidity effects of having two antigen-binding modules (Fabs) per antibody, antibodies may be able to crosslink CSP molecules on the sporozoite surface. Notwithstanding, transition between CSP topologies may be important for the infectious lifecycle of malaria, and therefore the antibodies described here and others may be useful probes to further understand its mechanism of action.

### Epitope Identification and Binding Stoichiometry.

The nsEM reconstructions indicate that up to nine copies of Fab311 are present on rsCSP, compared with only five for Fab317. From our epitope-mapping experiments, the crystal structure of the Fab311–peptide complex, and ITC affinity measurements, we established that the minimum Fab311 epitope consists of two NPNA repeats. Since the total number of NANP repeats in rsCSP is 19 with three interspersed NVDP repeats, that leaves a stretch of 16 NANP repeats or eight epitopes available for binding, which is slightly less than the nine Fabs that we observe in the nsEM map. There are two possible explanations for these observations. First, Fab311 can also bind with reasonable affinity to at least one of the NVDP repeats on CSP even though these sequences are not present on the RTS,S vaccine. If these NVDP repeats were fully available for antibody binding, the number of potential epitopes could increase to 11 [(19 NANP + 3 NVDP)/2]. A second possibility is that Fab311 can bind with reasonable affinity to single NANP repeats that are flanked by NVDP repeats. To further investigate the binding differences observed in the nsEM reconstructions between the antibodies, we performed ITC experiments to measure the binding of Fab311 and Fab317 to peptides that contained both NANP and NVDP repeats. Affinity measurements confirm that Fab311 has a reasonable affinity for the NVDP region, with *K*_d_s of 1.37 and 1.79 μM for the Ac-DPNANPNVDPNA-NH_2_ and Ac-NPNVDPNANPNV-NH_2_ peptides, respectively, compared with 0.45 and 12.09 μM for Fab317. Although Fab317 is able to bind the peptide tightly when the NVDP repeat is downstream from an NANP repeat, binding is abrogated when the order is reversed. Hence, the different binding stoichiometry for Fab311 and Fab317 can more likely be rationalized by the preferred minimum Fab317 epitope consisting of about three NPNA repeats and probably no binding to the NVDP repeat region. Thus, approximately five [(19 NANP – 3 NANP)/3] binding sites are available, which is exactly what we observe with nsEM.

### Implications for Vaccine Efforts and Injectable Biologics.

Our crystal structures show both similarities and differences in the way the same peptide is recognized by the two antibodies. The antibodies recognize the NPNA repeats as well-defined β-turns, modified turns, or as more extended structures. Additionally, nsEM reconstructions show that the NANP repeat region can be further stabilized by antibody interactions in the context of the rsCSP. These findings provide exciting opportunities for structure-assisted immunogen design. Since NMR data suggest that these type I β-turn conformations represent a major population of the NPNA repeats in solution, and since we observe NPNA type I β-turns in peptides bound to highly protective antibodies, stabilizing these secondary structure elements in next-generation CSP immunogens may elicit an improved antibody response. Indeed, attempts to stabilize type I β-turns in the NANP repeat go back to 1990 with covalent hydrogen-bond mimics (30). Five years later, the NPNA type I β-turn conformation was stabilized in solution by replacing proline with (S)-α-methylproline (31). Both approaches elicited polyclonal antisera in rabbits against the designed/synthetized peptides that recognized intact sporozoites. We propose that rational NANP-based immunogen design may start at the level of stabilizing the NPNA type I β-turns and gradually build in more complexity to mimic the superstructure of the repeat units that we observe by nsEM.

Finally, efforts are underway to determine if mAbs themselves can be used as interventions to prevent malaria parasite infection. To accomplish this goal, the mAbs will need to be optimized to achieve protection at the lowest possible serum concentration. The structures presented here provide a good starting point for further mAb engineering and for identifying mAbs with improved binding characteristics. As a single sporozoite is covered with hundreds of CSP molecules, which are continually shed (32), it is beneficial to use as little antibody as possible per sporozoite to target all sporozoites that enter the body. In this study, Fab317 would appear to be the more promising of the two antibodies in that regard. Further studies will assess whether other epitopes are available for both immunogen design and for antibody parsimony.

## Materials and Methods

Detailed material and methods are provided in *SI Materials and Methods*.

### Evaluation of the Protective Activity of mAbs.

mAbs 311 and 317 were administered to 7- to 8-wk-old C57BL/6 mice by i.v. injection before challenge with *P. berghei* chimeric sporozoites expressing *P. falciparum* CSP (3D7 strain) (22). Antibody inhibition of parasite development was analyzed by comparing the parasite liver burdens of experimental and naïve control mice. C57Bl/6 mice were housed in the animal facility of the Johns Hopkins Bloomberg School of Public Health. All animal procedures were approved by the Animal Care and Use Committee at Johns Hopkins University, protocol no. MO16H35.

### Protein Production.

Fab311 and Fab317 were expressed in FreeStyle 293F cells (Invitrogen) and purified using a lambda or kappa affinity column (GE Healthcare) followed by cation exchange chromatography (monoS). rsCSP was expressed in *Escherichia coli* (SHUFFLE cells; New England Biolabs) and purified as described (33). The rsCSP construct has three NVDP repeats interspersed with three NANP repeats followed by a continuous region of 16 NANP repeats.

### PepSpot Analysis.

A customized (NANP)_6_ peptide truncation array was purchased from JPT Peptide Technologies GmbH. Binding of Fab311 and Fab317 was visualized using HRP-conjugated anti-human Fab secondary antibodies.

### ITC.

ITC affinity measurements were performed using a MicroCal Auto-iTC200 instrument (GE Healthcare).

### X-Ray Crystallography.

Fab–(NPNA)_3_ peptide complexes were made by adding the peptide to the Fab in a 5:1 molar ratio. Crystals were obtained by sitting-drop vapor diffusion. X-ray diffraction data were collected at the Stanford Synchrotron Radiation Lightsource (SSRL) BL12-2 and the Advanced Photon Source (APS) BL23ID-B.

### nsEM.

rsCSP was saturated with Fabs and purified by size-exclusion chromatography. Complexes were imaged on a FEI Tecnai Spirit T12 electron microscope equipped with a Tietz TVIPS CMOS camera.

## Supplementary Material

Supplementary File
