# Supplementary material for: Structural basis for antibody recognition of the NANP repeats in Plasmodium falciparum circumsporozoite protein
Source: Proc Natl Acad Sci U S A. 2017 Nov 14;114(48):E10438–45. doi: 10.1073/pnas.1715812114 (PMC5715787; doi:10.1073/pnas.1715812114)
Supplement: Supplementary File [file pnas.201715812SI.pdf]

Supporting Information

Oyen et al. 10.1073/pnas.1715812114

SI Materials and Methods

General. All peptides were ordered from Innopep Inc. with a purity of >98% and contained chlorine counter ions. The peptides have N-terminal acetylation and C-terminal amidation to eliminate charges at the peptide termini. Fab genes were codon optimized, synthesized, and cloned into pHCMV3 vectors by GenScript. Antibody sequences have been identified from plasmablasts isolated from individuals in all groups receiving RTS,S vaccination in the Mal071 trial (21). Representative members of different antibody families from both regimens, as well as from protected and nonprotected individuals, have been isolated and sequenced (21). Antibodies 311 and 317 were selected for structural studies because of their functional activity, high avidity index, and similar heavy-chain germline genes.

Evaluation of the Protective Activity of mAbs. mAbs (100 μg for Ab311 and 300 μg for Ab317 and Ab2A10) against the NANP repeats of *P. falciparum* CSP were administered to 7- to 8-wk-old C57BL/6 mice (five mice per group) by i.v. injection [in 200 μL of PBS (pH 7.4)]. The mice were challenged 5–10 min later by i.v. injection with 2,000 *P. berghei* chimeric sporozoites expressing *P. falciparum* CSP (3D7 strain) generated as previously described (22). Forty hours later, livers were harvested, and RNA was isolated to quantify the malaria-specific 18s rRNA levels by real-time PCR (34). Inhibition of parasite development was calculated by comparing the parasite liver burdens of experimental and naive control mice. The statistical analyses were done with the Mann–Whitney *U* test. Data were plotted using GraphPad Prism 6.0 software. No samples were excluded from statistical analyses.

Expression and Purification of Recombinant CSP. The rsCSP construct of the 3D7 strain of *P. falciparum* was expressed and purified as previously described (33). In short, a pET28a plasmid containing the rsCSP construct was used to transform *E. coli* SHUFFLE competent cells (New England Biolabs). A single colony was used to start a 50-mL overnight culture. Two 1-L cultures were inoculated the next day with 25 mL overnight culture and were grown at 37 °C. When the optical density at 600 nm reached a value of 1, the cultures were induced with 1 mM isopropyl β-D-1-thiogalactopyranoside for 6 h. The cells then were harvested and lysed by microfluidization. The lysate was incubated overnight with Ni cOmplete resin (Roche) and was eluted using a buffer containing 200 mM imidazole. Typically, 10 mg of protein was obtained per 2 L of culture. Samples for EM were made by mixing rsCSP with Fab at a 1:10 molar ratio. The complex was purified using size-exclusion chromatography in Tris-buffered saline (TBS) buffer (50 mM Tris·HCl, 137 mM NaCl, 2.7 mM KCl, pH 8.0).

PepSpot Analysis. To determine the minimal NANP repeat sequence for binding, we designed a custom peptide array (PepSpot) that was purchased from JPT Peptide Technologies, GmbH. The array consists of a series of truncated peptides derived from a $(NANP)_6$ peptide, which are spot-synthesized onto a cellulose membrane to assess binding of anti-CSP Fab fragments. The membrane was rinsed with methanol for 5 min and then was washed three times for 3 min each with TBST buffer [50 mM Tris·HCl, 137 mM NaCl, 2.7 mM KCl, 0.05% (vol/vol) Tween 20, pH 8.0]. The membrane then was blocked with blocking buffer [5 % (wt/vol) bovine serum albumin in TBST) and was incubated at room temperature for 2 h. Following the 2-h incubation, the membrane was incubated for 3 h at room temperature with a 10-μg/mL solution of the Fab of interest in blocking buffer. The membrane was then washed three times for 5 min each in TBST buffer, after which it was incubated for 2 h with an anti-human Fab secondary antibody conjugated to HRP (Sigma-Aldrich) at a 1:20,000 dilution in blocking buffer. After the membrane was washed three times with TBST buffer for 5 min each, the secondary antibody was visualized by incubating the membrane in SuperSignal West Pico Chemiluminescent Substrate (Thermo Fischer) and exposing it for 1–2 s using the Bio-Rad ChemiDoc XRS+ system.

ITC. A MicroCal Auto-iTC200 (GE Healthcare) was used to perform ITC measurements. Before the experiments, all proteins were extensively dialyzed against Dulbecco's PBS (Thermo Fischer). The peptides were placed in the syringe at a concentration of 122 μM for Ac-NPNANPNA-NH_2, 116 μM for Ac-NPNANPNANPNA-NH_2, 89 μM for Ac-NPNVDPNANPNV-NH_2, and 110 μM for Ac-DPNANPNVDPNA-NH_2, whereas the concentrations of Fab fragments in the cell were 10.3 and 9.7 μM for Fab311 and Fab317, respectively. The Fab and peptide concentrations were determined by UV absorbance at 280 nm and 205 nm. Molar extinction coefficients for the peptides were estimated using a previously published method (35). Experiments were carried out in triplicate at 25 °C, except for Fab317 binding to Ac-NPNVDPNANPNV-NH_2, which was done in duplicate, and consisted of 16 injections of 2.45 μL each, with injection duration of 4.9 s, injection interval of 180 s, and reference power of 5 μCal. Fitting of the integrated titration peaks was performed with Origin 7.0 software using a single-site binding model. The first data point and any outliers were excluded from the fit.

X-Ray Crystallography. The Fab311–peptide and Fab317–peptide complexes were crystallized from solutions containing Fab311 or Fab317 at 11 mg/mL in TBS buffer (50 mM Tris·HCl, 137 mM NaCl, 2.7 mM KCl, pH 8.0) with a 5:1 molar ratio of $(NPNA)_3$ peptide to Fab. Crystals were grown using sitting-drop vapor diffusion with a well solution containing 0.1 M MES (pH 6.0), 36% PEG400, 5% PEG3000 for the Fab311–peptide complex, and 0.1 M sodium citrate (pH 4.0), 1 M lithium chloride, 20% PEG6000 for the Fab317–peptide complex. Crystals were grown at 298 K and typically appeared within 3 d. Fab311–peptide crystals were cryo-cooled without additional cryoprotection, while Fab317–peptide crystals were cryoprotected by soaking in a well solution supplemented with 30% ethylene glycol. X-ray diffraction data were collected at the SSRL BL12-2 for the Fab317–peptide complex and the APS beamline 23ID-B for the Fab311–peptide complex. Data collection and processing statistics are outlined in Table S1. Datasets were indexed, integrated, and scaled using the HKL-2000 package (36). The structures were solved by molecular replacement using PHASER (37) with a homology model [SWISS-MODEL (38–40) and PIGSPro (41)] for either Fab311 or Fab317 as a search model. After refinement of the Fab using phenix.refine (42) combined with additional manual building cycles in Coot (43), clear Fo-Fc density was observed in the Fab-combining site for the peptide. The peptide was manually built into the difference density Fo-Fc map, followed by additional rounds of refinement of the complex in phenix.refine (42) and manual building cycles in Coot (43).

BSAs were calculated with the program MS (44) using a 1.7-Å probe radius and standard van der Waals radii (45). Dihedral

angles and rmsds were calculated using ANGLES (CCP4) and SUPERPOSE (46), respectively. Hydrogen bonds were evaluated with the program HBPLUS (47). Pearson correlation coefficients were calculated using the scipy.stats module in Python. Ramachandran plots were generated in R using the previously reported ϕ, ψ angle distribution (48).

Single-Particle nsEM. Purified complexes (as described above) at 10 mg/mL were diluted to 0.2 mg/mL with TBS buffer, deposited onto glow-discharged carbon-coated copper-mesh grids, and stained with 2% uranyl formate. An FEI Morgagni (80 keV) electron microscope aided in screening the grids for correct particle density and stain thickness. All data were collected on an FEI Tecnai Spirit T12 (120 keV) electron microscope with a Tietz TVIPS CMOS (4K × 4K) camera and Leginon software (49) and were stored/processed within the Appion database (50). Images were collected at 52,000× magnification and had a defocus of −1.50 µm and a pixel size of 2.05 Å per pixel. Particles were picked from raw micrographs and placed into boxes of 160 × 160 pixels using the DoGpicker software (51) and compiled into a stack. Particles were then aligned with iterative multivariate statistical analysis/multireference alignment (MSA/MRA) (52). Class averages were cleaned up by removing classes with double particles or obvious contaminants. The clean stack was then used as the input for another round of 2D classification, 3D classification, and 3D refinement using RELION 2.0 software (53). For rsCSP–Fab317, the clean stack was also the input for the CryoSPARC software (54), which outputted a superior model than RELION 2.0. There were 6,320 particles for the rsCSP–Fab311 complex and 38,361 particles for the rsCSP–Fab317 complex that went into the 3D refinement. Fab311 and Fab317 were taken from the crystal structures with $(NPNA)_3$ peptides removed, low-pass filtered to 30 Å, and were fit into the features corresponding to the Fabs in the respective nsEM maps of the Fab–rsCSP complex (Fig. S4) using Chimera (University of California, San Francisco) (53). After each fit, a correlation value was calculated in which a perfect fit was 1. For each of the Fab311 and Fab317 complexes, all Fab fits had correlation values of 0.96. The crystal structure of the peptide–Fab complex was then docked into the low-pass–filtered fitted Fab densities to obtain the approximate location of the $(NPNA)_3$ peptides. A cylinder was fitted to the docked peptides for the rsCSP–Fab311 complex using David Eberly's method (https://www.geometrictools.com/Documentation/CylinderFitting.pdf) implemented in Python.

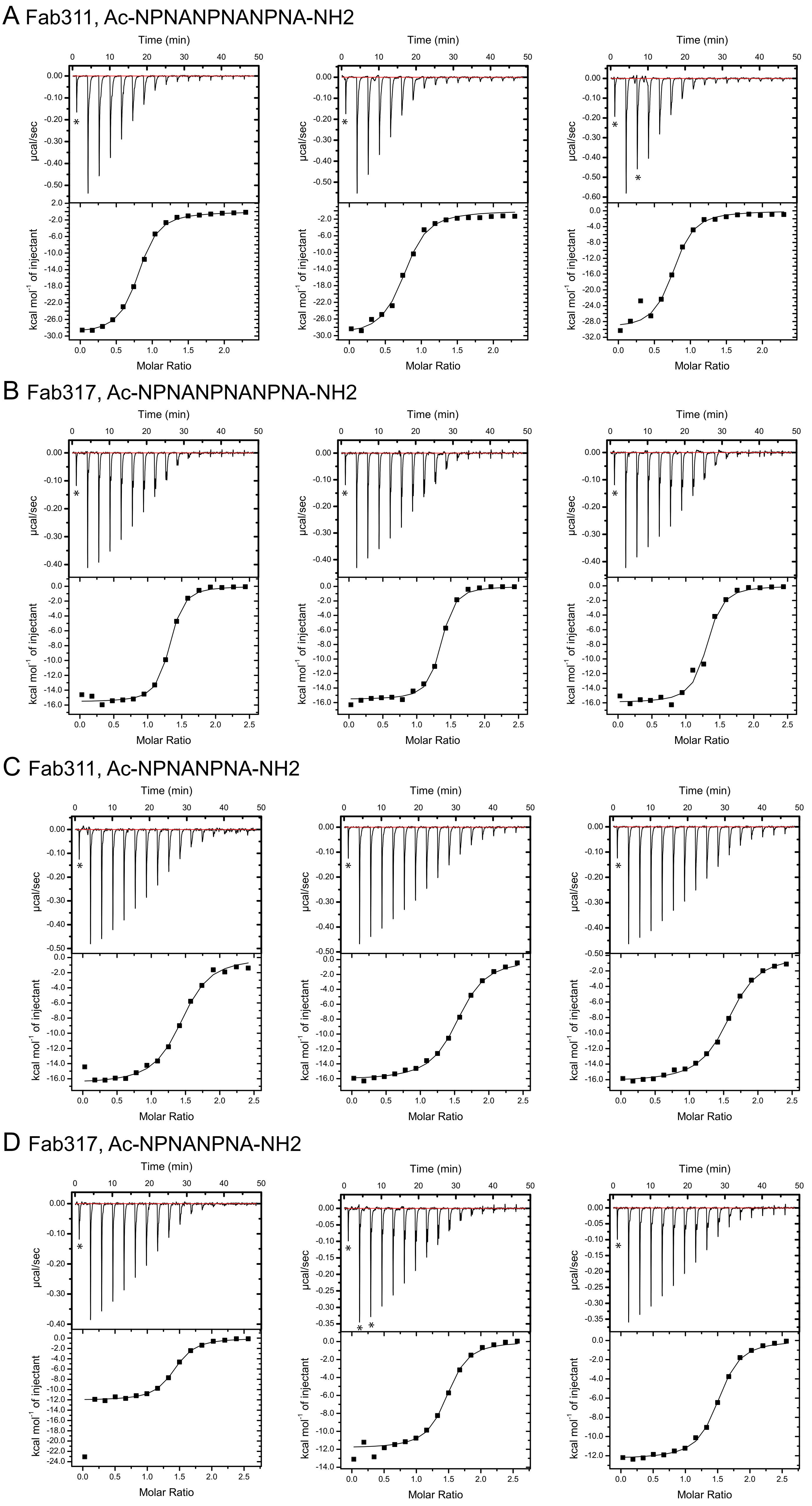

Fig. S1. ITC binding curves for NANP repeat peptides binding to Fab311 and Fab317. ITC binding data for the 12-mer peptide Ac-NPNANPNANPNA-NH_2 to Fab311 (*A*) and Fab317 (*B*) and for the 8-mer peptides Ac-NPNANPNA-NH_2 to Fab311 (*C*) and Fab317 (*D*), respectively. Data points not included in the fit are indicated by an asterisk.

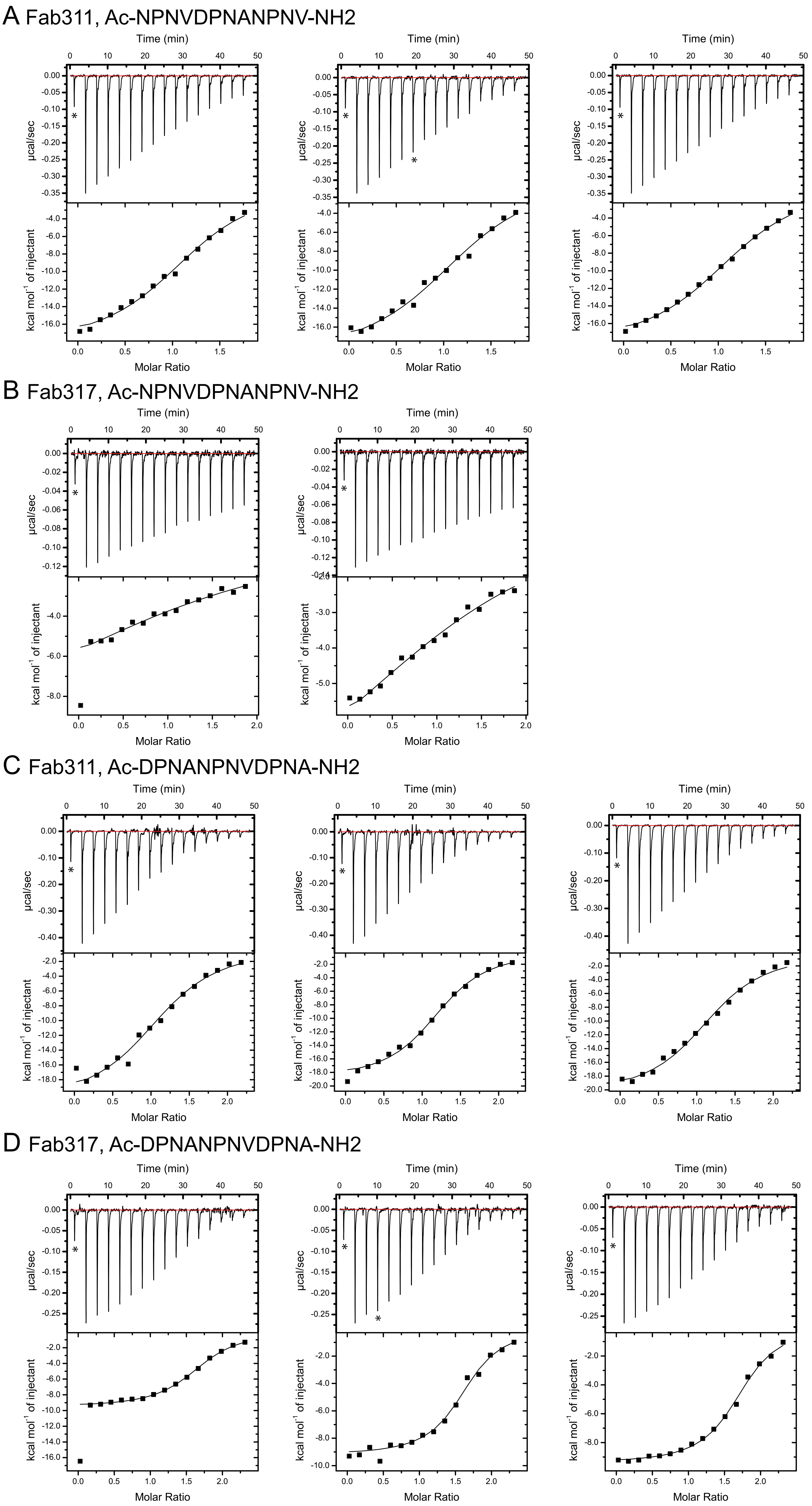

Fig. S2. ITC binding curves for NVDP-containing repeat peptides binding to Fab311 and Fab317. ITC binding data for the 12-mer peptide Ac-NPNVDPNANPNV-NH_2 to Fab311 (*A*) and Fab317 (*B*) and for the 12-mer peptides Ac-DPNANPNVDPNA-NH_2 to Fab311 (*C*) and Fab317 (*D*), respectively. Data points not included in the fit are indicated by an asterisk.

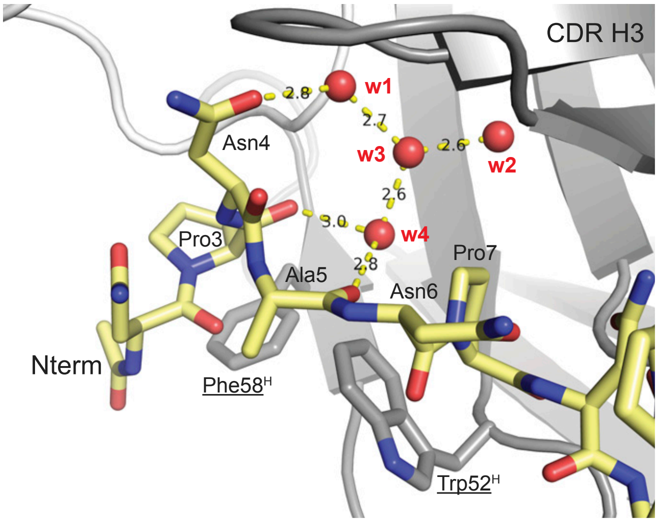

Fig. S3. Buried waters and CH/π interactions at the binding interface of the Fab311–peptide. Interfacial waters w1 and w4 engage in hydrogen bonding with N-terminal–associated peptide residues Pro3, Asn4, and Ala5 and with other water molecules w2 and w3. Pro3 and Pro7 are engaged in CH/π interactions with Phe58^{H} and Trp52^{H}, respectively. The peptide is show in stick representation (yellow carbons), and the Fab is shown in cartoon representation (light chain in light gray and heavy chain in dark gray). The N terminus of the peptide and CDR H3 are indicated in the figure. Underlined residue numbers belong to the Fab.

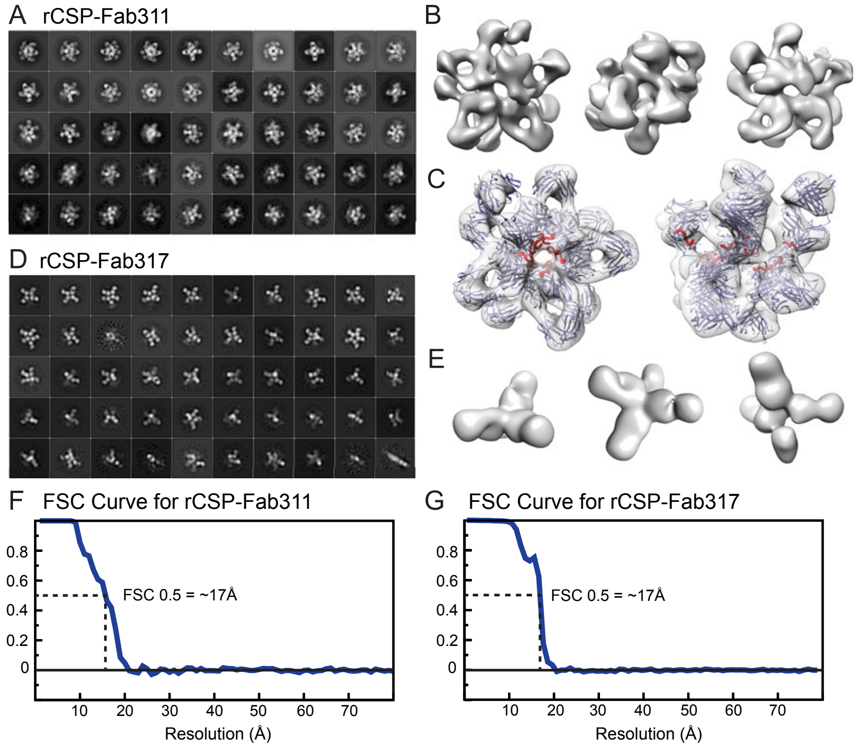

Fig. S4. 2D class averages, model views, and Fourier shell correlation curves. Full panel of representative 2D class averages, multiple views of the 3D reconstructions, and the FSC curves for the rsCSP–Fab311 (*A*, *B*, and *F*) and rsCSP–Fab317 (*D*, *E*, and *G*) complexes. The calculated resolution for both models is 17 Å. (*C*) Fits of the Fab311–(NPNA)$_3$ complexes in the negative stain map (front and side view). The Fabs are shown in cartoon representation (blue), and the peptides are shown as red tubes.

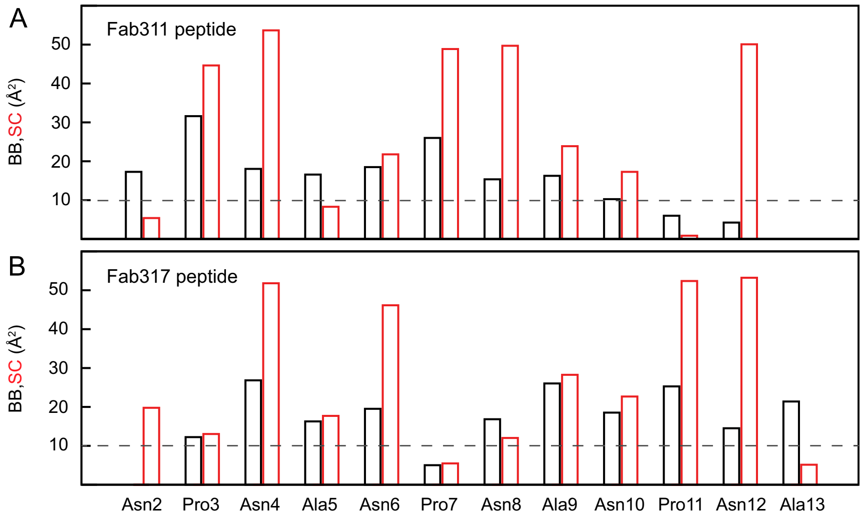

Fig. S5. BSAs for peptides bound to Fab311 and Fab317. BSAs per residue for the main-chain (BB, black) and side-chain (SC, red) atoms are plotted for the peptide bound to Fab311 (*A*) and Fab317 (*B*).

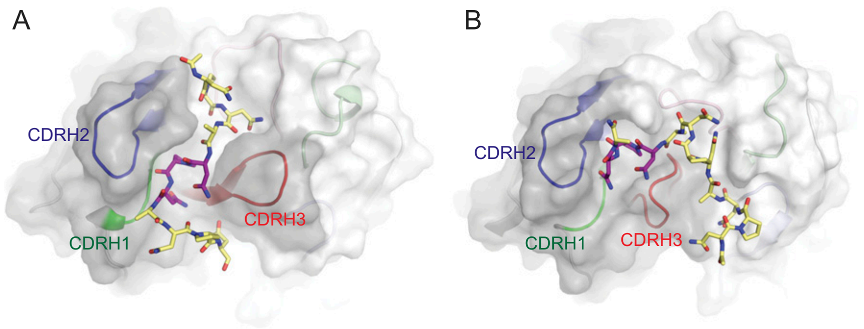

Fig. S6. Orientation of highly similar NPN units in Fab311 and Fab317 complexes with the $(NPNA)_3$ peptide. Paratope representation for Fab311 (*A*) and Fab317 (*B*) with a transparent dark gray surface for the heavy chain and a transparent light gray surface for the light chain. The underlying CDR loops are shown in cartoon representation and are colored green (H1), blue (H2), red (H3), light green (L1), light blue (L2), and pink (L3). The $(NPNA)_3$ peptide is shown in stick representation (yellow carbons). NPN units that share an identical orientation relative to the CDR loops are colored purple and represent residues 6–8 for Fab311 and residues 10–12 for Fab317.

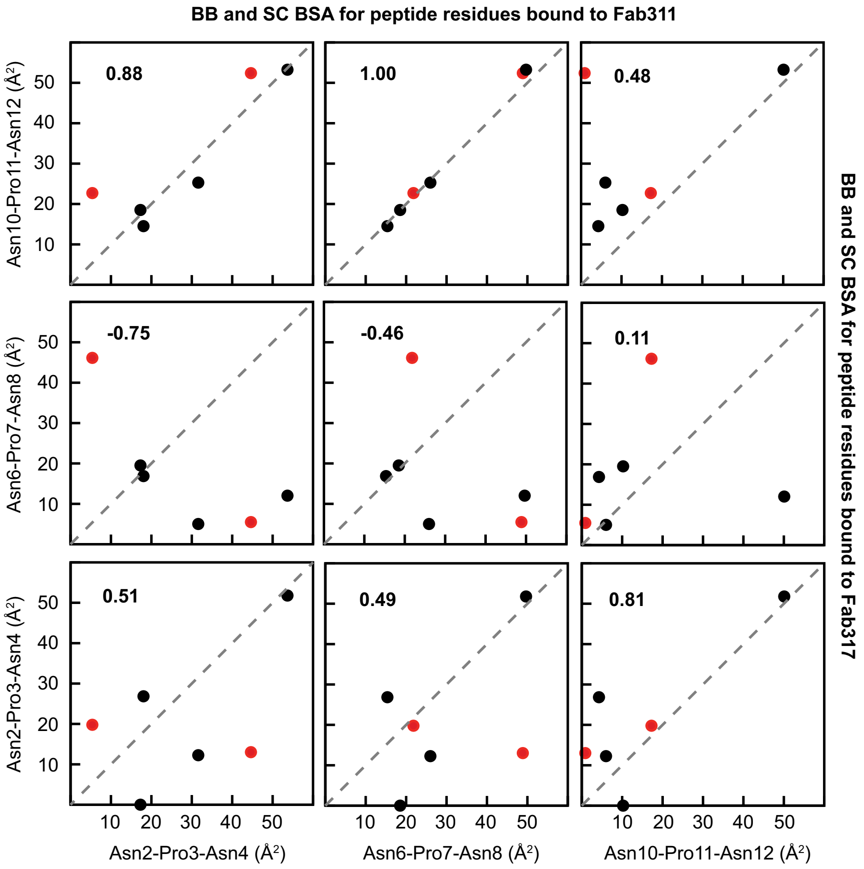

Fig. S7. Correlations of backbone (BB) and side-chain (SC) BSAs. BSA correlation plots are shown between the NPN units of the Fab311-bound peptide versus the Fab317-bound peptide for both backbone and side-chain atoms. Pearson correlation coefficients are given for each correlation plot. The BSAs from the Fab311-bound peptide residues Asn6, Pro7, and Asn8 show a perfect linear correlation (Pearson correlation coefficient of 1.00) with the BSAs from the Fab317-bound peptide residues Asn10, Pro11, and Asn12.

Table S1. X-ray data collection and refinement statistics

Data parameter	Fab311–$(NPNA)_3$	Fab317–$(NPNA)_3$
Data collection		
Beamline	APS 23ID-B	SSRL 12–2
Wavelength, Å	1.03320	0.97946
Space group	$P2_12_12_1$	$P2_1$
Unit cell parameters, Å, °	a = 66.12, b = 44.73, c = 186.08	a = 82.66, b = 65.34, c = 99.04, β = 106.40
Resolution, Å	50.00–2.10 (2.14–2.10)*	50.00–2.40 (2.44–2.40)*
Unique reflections	32,026 (1,572)*	38,939 (1,956)*
Redundancy	5.1 (4.1)*	6.1 (6.3)*
Completeness, %	98.1 (97.6)*	97.8 (98.6)*
$<I/\sigma_I>$	11.0 (1.4)*	11.2 (2.7)*
R_{sym}†, %	17.4 (92.1)*	18.1 (102)*
R_{pim}†, %	7.7 (50.0)*	7.9 (43.0)*
$CC_{1/2}$‡, %	82.1 (46.5)*	92.0 (73.2)*
Refinement statistics		
Resolution, Å	46.52–2.10	40.93–2.40
Reflections (work)	30,360	37,031
Reflections (test)	1,606	1,882
R_{cryst}§/R_{free},¶ %	20.4/23.6	17.6/21.7
No. of atoms		
Protein	3,351	6,705
Water	242	485
Buffer components	53	0
Average B-value, $Å^2$		
Fabs	38	36
Peptide	47	36
Water	45	40
Buffer components	119	0
Wilson B-value, $Å^2$	32	29
Rmsd from ideal geometry		
Bond length, Å	0.002	0.003
Bond angle, °	0.67	0.59
Ramachandran statistics#		
Favored, %	96.73	96.54
Outliers, %	0.00	0.00

*Numbers in parentheses refer to the highest resolution shell.

†$R_{sym} = \Sigma_{hkl} \Sigma_i \mid I_{hkl,i} - <I_{hkl}> \mid / \Sigma_{hkl} \Sigma_i I_{hkl,i}$ and $R_{pim} = \Sigma_{hkl} (1/(n-1))^{1/2} \Sigma_i \mid I_{hkl,i} - <I_{hkl}> \mid / \Sigma_{hkl} \Sigma_i I_{hkl,i}$, where $I_{hkl,i}$ is the scaled intensity of the ith measurement of reflection h, k, l, $<I_{hkl}>$ is the average intensity for that reflection, and n is the redundancy.

‡$CC_{1/2}$ = Pearson correlation coefficient between two random half datasets.

§$R_{cryst} = \Sigma_{hkl} \mid F_o - F_c \mid / \Sigma_{hkl} \mid F_o \mid \times 100$, where F_o and F_c are the observed and calculated structure factors, respectively.

¶R_{free} was calculated as for R_{cryst} but on a test set comprising 5% of the data excluded from refinement.

#From MolProbity (55).

Table S2. Hydrogen-bonding interactions of the $(NPNA)_3$ peptide bound to Fab311 and Fab317

Fab atom	Peptide atom	Peptide	Waters	Distance, Å
Peptide bound to Fab311				
Pro3-O	Ser95C^{L}-OG			2.75
Pro3-O			w4	3.03
Asn4-O	Tyr97^{H}-OH			2.52
Asn4-ND2	Thr100^{H}-O			3.05
Asn4-OD1			w1	2.84
Asn4-N		Asn2-OD1		3.21
Ala5-O			w4	2.77
Pro7-O	Tyr52A^{H}-N			3.17
Asn8-OD1	Gly33^{H}-N			2.78
Asn8-ND2	Ala95^{H}-O			2.93
Asn8-N		Asn6-OD1		2.88
Ala9-N	Asn31^{H}-O			2.96
Asn12-OD1	Tyr97^{H}-N			2.73
Peptide bound to Fab317				
Asn4-ND2	Ser49^{L}-OG			2.62
Asn4-O	Tyr91^{L}-OH			2.51
Asn4-N		Asn2-OD1		2.58
Ala5-N		Asn2-O		3.22
Asn6-ND2	Tyr91^{L}-O			2.87
Asn6-N	Ser99^{H}-OG			2.70
Asn8-N		Asn6-OD1		2.95
Ala9-O	Ser100^{H}-OG			2.94
Ala9-N		Asn6-O		3.04
Pro11-O	Asn56^{H}-ND2			3.12
Asn12-OD1	Tyr52A^{H}-N			2.96
Asn12-N		Asn10-OD1		2.88
Ala13-N		Asn10-O		3.03
